# Clinical Outcome and Prognostic Factors in Childhood Acute Lymphoblastic Leukemia—A Retrospective Cohort Study of Patients from Southern Mexico

**DOI:** 10.3390/jcm15176758

**Published:** 2026-08-31

**Authors:** Francisca Morales-Vital, Diego Moreno-Loaeza, Victor Manuel Alvarado-Castro, Jennifer Meza-Miranda, César López-Camarillo, Rael Escoto-Hernández, Luis Alberto Ojeda-Campos, Olga Lilia Garibay-Cerdenares, Azucena Ocampo-Bárcenas, Eloísa Ibarra-Sierra

**Affiliations:** 1 Research Department, Dr. Arturo Beltrán Ortega State Cancer Institute, Acapulco 39610, Guerrero, Mexico; franciscavital3025@gmail.com (F.M.-V.); tadeo.1107@gmail.com (D.M.-L.); frata20@hotmail.com (J.M.-M.); direccion.general@cancerologiagro.gob.mx (R.E.-H.); ojedacampos@hotmail.com (L.A.O.-C.); ocampobarcenas80@gmail.com (A.O.-B.); 2 Center for Research of Tropical Diseases, Autonomous University of Guerrero, Acapulco 39640, Guerrero, Mexico; vmalvarado@uagro.mx; 3 Genomics Sciences Program, Autonomous University of Mexico City, Mexico City 03100, Mexico; cesar.lopez@uacm.edu.mx; 4 Secihti-Autonomous University of Guerrero, Chilpancingo 39090, Guerrero, Mexico; olgaribayce@secihti.mx

**Keywords:** acute lymphoblastic leukemia, prognostic factors, outcome, overall survival, relapse, Kaplan–Meier, treatment non-adherence

## Abstract

**Background/Objectives**: B-cell acute lymphoblastic leukemia (B-ALL) remains the most common pediatric malignancy. This study aimed to evaluate clinical outcomes in pediatric B-ALL in Southern Mexico and determine how socioeconomic vulnerabilities—specifically indigenous status and treatment non-adherence—independently drive relapse and compromise survival in a resource-limited setting. **Methods**: A retrospective cohort study was conducted, including 146 patients (0–17 years) treated between 2010 and 2024 using modified Total XIII and XV protocols. Survival was estimated via Kaplan–Meier. Independent prognostic factors for overall survival (OS) and relapse were identified using multivariable Cox proportional hazards models. **Results**: The 5-year OS was 50%, and event-free survival (EFS) was 37.6%. Survival varied significantly by clinical risk: 88% for low-risk, 77% for standard-risk, and 31% for high-risk patients. Relapse occurred in 45.8% of patients. Beyond established biological predictors like hyperleukocytosis and high-risk cytogenetics, socioeconomic vulnerabilities profoundly impacted outcomes. Documented treatment non-adherence significantly reduced 5-year OS (23.4% vs. 56.1%, *p* = 0.001) and disproportionately affected indigenous patients (55.5% vs. 10.1% in non-indigenous). Multivariable analysis identified indigenous status as an independent predictor of increased relapse hazard (HR = 1.89, *p* = 0.049), alongside male sex and high leukocyte count. **Conclusions**: Survival outcomes in this cohort are markedly lower than in high-income countries. The high incidence of relapse is deeply intertwined with local structural inequities. Improving survival requires complementing biological risk stratification with targeted strategies to overcome socioeconomic barriers and prevent treatment non-adherence.

## 1. Introduction

Acute lymphoblastic leukemia (ALL) is a genetically heterogeneous group of hematological malignancies characterized by uncontrolled proliferation of immature lymphoid cells [1]. According to GLOBOCAN 2022, leukemia persists as one of the leading causes of cancer mortality worldwide, with ALL standing out due to its predominance in children and its high incidence in Latin America and Asia [2]. Based on immunophenotype, acute lymphoblastic leukemia (ALL) can be classified as B-ALL and T-ALL, representing 85% and 15% of cases, respectively [3], with similar results with modern therapy [4].

The development of new therapeutic agents in conjunction with the implementation of highly accurate diagnostic technologies has contributed to improving the survival rate to nearly 90% in patients with childhood ALL treated in high-income countries [5,6]. However, in low- and middle-income countries, the decline in survival is considerable, reaching estimated survival rates of around 50% in countries like Mexico [5]. While biological risk stratification remains the standard of care, survival in resource-limited settings is profoundly shaped by structural barriers and socio-economic disparities. Factors such as delayed diagnosis, lack of pediatric intensive care units (PICUs), limited supportive care resources, and treatment non-adherence critically compromise the successful implementation of contemporary intensive chemotherapy regimens.

The diagnosis and subsequent administration of chemotherapy depend on the patient’s stratified risk classification. This is established by combining clinical indicators at diagnosis (e.g., age, with cutoffs at 1–9.9 years vs. <1 or ≥10 years; white blood cell (WBC) counts < 50 × 10^9^ L vs. ≥50 × 10^9^/L) and the findings of a minimal residual disease (MRD) assay to evaluate treatment response [6]. In addition to these clinical parameters, the identification of genetic abnormalities in ALL is crucial for implementing targeted therapies and personalized primary therapy adjustment, leading to better disease management [1]. While specific cytogenetic alterations are globally recognized as prognostic drivers—such as the ETV6-RUNX1 fusion conferring a favorable prognosis, versus high-risk alterations like t(9;22)(q34;q11.2) and KMT2A rearrangements [7,8]—the prognostic weight of these established biological factors must be contextualized within the operational realities of local healthcare systems.

In Mexico, information on the prognosis of pediatric ALL is very scarce; this data limitation hinders an accurate assessment of the efficacy of currently implemented diagnostic and therapeutic strategies. The primary objective of this study was to analyze the overall survival of patients with B-ALL, focusing on risk groups. The secondary objective was to evaluate not just established biological factors, but also how socioeconomic and demographic vulnerabilities—specifically indigenous status, geographic region of origin, and treatment non-adherence—independently influence overall survival (OS) and relapse, moving beyond conventional biological risk stratification in a cohort treated at a secondary-level oncological center in Southern Mexico. Ultimately, our findings suggest that the low overall survival and high relapse rates observed in this cohort are deeply intertwined with local vulnerabilities, underscoring the urgent need to complement clinical risk stratification with targeted strategies that overcome socio-economic barriers and prevent non-adherence to treatment.

## 2. Materials and Methods

### 2.1. Study Design and Population

This was a retrospective cohort study that included 146 patients aged 0–17 years, diagnosed with B-cell precursor ALL, who were treated and followed up at the Pediatric Oncology Unit of the Dr. Arturo Beltrán Ortega State Cancer Institute (IECAN) in Acapulco, Guerrero, Mexico. Inclusion criteria were: (1) patients aged 0–17 years, and (2) patients newly diagnosed with B-cell ALL. Exclusion criteria were: (1) phenotypes other than B-cell ALL, or (2) having another pathology. Elimination criteria were: (1) patients who permanently discontinued treatment and did not return for follow-up, or (2) patients with incomplete medical records that prevented assessment of the study outcomes. Temporary treatment interruptions followed by return to treatment were not considered an elimination criterion.

### 2.2. Diagnosis

Before initiating treatment, patients underwent a detailed baseline evaluation that included medical history, physical examination and hematological analyses for white blood cell count and morphological characterization. Immunophenotyping was performed by flow cytometry using bone marrow aspirates collected in tubes containing EDTA. Additionally, the presence of 28 leukemia-associated translocations was assessed by nested multiplex RT-PCR using the Hemavision 28N kit (HV01-28N), DNA Diagnostic A/S, Risskov, Denmark.

### 2.3. Risk Group Classification and Treatment Strategy

The treatment protocol was based on stratifying patients into risk groups. Risk group classification was performed initially or after remission-induction chemotherapy according to the criteria presented in Table 1, allowing for uniform management of the groups. Treatment was administered according to current guidelines, using modified versions of the St. Jude Total XIII treatment protocol between January 2010 and December 2020, and the modified St. Jude Total XV protocol between January 2021 and December 2024 (the full adapted protocols are provided in Supplementary Files S1 and S2). Although patients were treated according to either the St. Jude Total XIII or Total XV protocols, the subgroup receiving the Total XV regimen was too small (*n* = 17) to permit a sufficiently powered statistical comparison of survival outcomes between the two treatment groups.

Patients with BCR::ABL1-positive disease received tyrosine kinase inhibitors in addition to standard chemotherapy.

CNS-directed therapy: Prophylaxis was initiated prior to systemic treatment with an age-adjusted dose of intrathecal (IT) cytarabine (40 mg for 1–2 years; 50 mg for 2–3 years; 60 mg for >3 years). During the consolidation phase, high-dose methotrexate (2.5 g/m^2^ for low risk; 5 g/m^2^ for standard/high risk) was administered as a 24-h infusion, accompanied by leucovorin rescue. Concurrently, triple intrathecal chemotherapy (methotrexate, cytarabine, and hydrocortisone) was administered on days 1, 15, 29, and 43. Additional intensive intrathecal therapy (twice a week for 2 weeks) was strictly reserved for high-risk CNS presentations, such as CNS-2/3 status (CNS-2, <5 leukocytes/μL with evidence of blasts on cytocentrifuge examination), traumatic lumbar puncture, hyperleukocytosis (>50,000/µL), or the presence of t(9;22).

### 2.4. Sociodemographic and Clinical Compliance Variables

To evaluate the impact of structural and behavioral factors beyond tumor biology, specific sociodemographic and clinical compliance variables retrieved from medical records were analyzed:

Indigenous status: Patients were classified as having indigenous status if medical records documented an indigenous language as their primary or sole language of communication, with Spanish spoken only as a secondary language or not spoken at all.

Geographic region: Patient origin was categorized based on their administrative geographic region of residence within the State of Guerrero.

Treatment non-adherence: This variable was defined as instances where patients failed to report to the hospital on the specific dates scheduled by the attending physician for chemotherapy administration. Typically, these patients did present for treatment, but with intermittent delays or postponements past their designated appointment dates.

### 2.5. Relapse

The impact of relapse on survival was evaluated, defining relapse as the return of disease in patients who had achieved complete remission (<5% blasts, no extramedullary disease) with first-line therapy. Bone marrow relapse was defined as ≥25% morphological blasts or ≥5% blasts with concomitant extramedullary relapse, and CNS relapse as ≥5 leukocytes/microliter of cerebrospinal fluid, with blasts on cytocentrifugation, confirmed by biopsy. Relapses were classified by timing: very early (<18 months), early (≥18–<36 months), and late (≥36 months) from initial diagnosis. Minimal Residual Disease (MRD) was assessed at the end of induction (day 28) using flow cytometry with a sensitivity of 10^−4^. Additionally, the presence of relapse to the Central Nervous System (CNS) was assessed through cytological analysis of cerebrospinal fluid.

### 2.6. Statistical Analysis

Statistical analysis was performed using the R statistical language version 4.5.0 [9]. The tidyverse package was used for data cleaning and transformation. Survival analysis and proportional hazards model fitting were performed using the survival package. The survminer package was used to visualize survival curves. Categorical variables were described using frequencies and percentages. Continuous variables were summarized using measures of central tendency and dispersion according to their distribution. Overall survival was estimated using the Kaplan–Meier method, using the time from the start of treatment to death or the last available follow-up. Event-free survival (EFS) was defined as the time from diagnosis to the first occurrence of relapse or death from any cause. Patients without either event were censored at the date of last follow-up. EFS was estimated using the Kaplan–Meier method, and survival probabilities were calculated with 95% confidence intervals. In addition, the cumulative incidence of relapse was estimated using a competing-risk approach, considering death without relapse as the competing event. Cumulative incidence estimates with 95% confidence intervals (95% CI) were calculated at 2 and 5 years. Patients who did not experience the event at the end of follow-up were considered censored. Survival curves were compared between groups using the log-rank test. Differences in survival were assessed according to sex, age group, initial white blood cell count, number of relapses, and type of cytogenetic translocation. Cumulative survival at 5 years was estimated using Kaplan–Meier curves with their respective 95% confidence intervals.

To identify independent prognostic factors, multivariable Cox proportional hazards regression analyses were performed for two clinical outcomes: overall survival and relapse. Overall survival was defined as the time from diagnosis to death from any cause, with surviving patients censored at the date of last follow-up. For the relapse analysis, the time from diagnosis to the first documented relapse was considered the event time, and patients without relapse were censored at their last follow-up. The same baseline covariates (sex, age group, leukocyte count at diagnosis, translocation status, clinical risk group, geographic region, indigenous status, and treatment non-adherence) were included in both multivariable models. Hazard ratios (HRs) with 95% confidence intervals were estimated, and statistical significance was established at *p* < 0.05.

Ethical declaration: The study protocol was approved by the Ethics and Research Committee of IECAN (Registration CONBIOÉTICA-12-CEI-001-20190726, code DGIECAN/CCHGC/CEI/PRO-041-2024). Given its retrospective nature, the committee waived the requirement for informed consent. Information was collected from medical records. The procedures were performed in accordance with the principles of the Declaration of Helsinki and in compliance with NOM-012-SSA3-2012 for research involving human subjects.

## 3. Results

### 3.1. Patient Characteristics

A total of 146 patients diagnosed with B-ALL between January 2010 and December 2024 were included in the study. Table 2 summarizes the general characteristics of the entire cohort. The median age of the patients at diagnosis was 8 years (IQR, 4–13 years). Seventy-four patients (50.7%) were male, while 72 (49.3%) were female. Regarding sociodemographic profile and treatment compliance, 27 patients (18.4%) had officially documented non-adherence to the prescribed chemotherapy protocol, and 18.5% belonged to an indigenous group. Concerning the patients’ place of origin, most were from the Acapulco region (22.6%), followed by the Costa Chica region (21.2%). At the end of the study, 76 (52.1%) patients were alive, while the remaining 70 (47.9%) had died. Out of the deceased patients, 42 (60%) died due to disease progression, relapse, or refractory leukemia. Documented Treatment-Related Mortality (TRM)—including severe infections (e.g., Klebsiella pneumoniae sepsis, pneumonia) and chemotherapy toxicities (e.g., neutropenic colitis, heart failure)—accounted for 7 cases (10%). Two patients (2.8%) died from secondary neoplasms. Crucially, for 19 patients (27.1%), the exact terminal medical cause of death was unavailable in the clinical records, largely reflecting patients who discontinued treatment and passed away in their rural communities. Regarding the patients’ place of origin, most were from the Acapulco region (22.6%), followed by the Costa Chica region (21.2%).

### 3.2. Biological and Clinical Prognostic Factors

**Figure 1:**
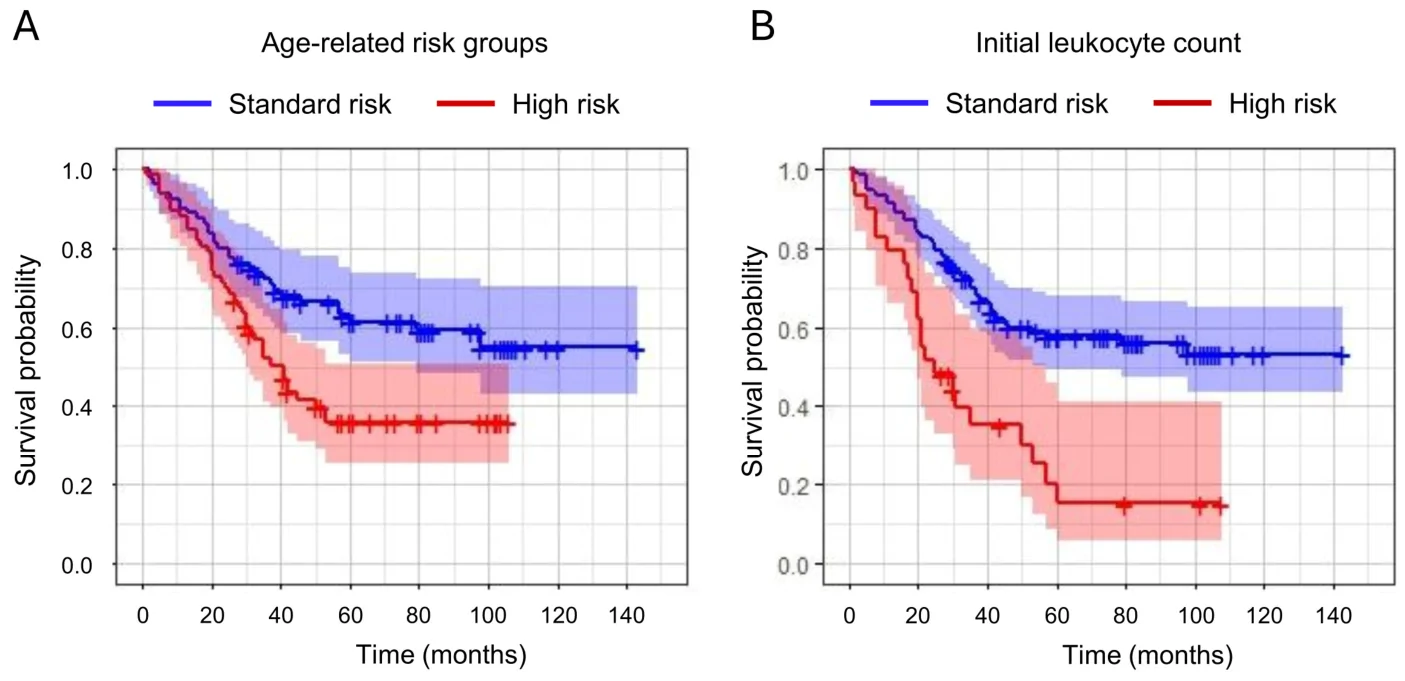
Kaplan–Meier estimates of ALL overall survival rates according to (**A**) age-related risk groups [standard risk (1–9.9, blue line); high risk (<1, ≥10, red line)] and (**B**) initial leukocyte count [standard risk (<50,000, blue line), *p* = 0.0075; high risk (≥50,000, red line)]. *p* < 0.0001.

As a first approach, the impact of sex, age, and white blood cell count (WBC) at diagnosis on overall survival was evaluated. Kaplan–Meier analysis showed a trend toward lower overall survival (OS) in male patients compared to female patients; however, these differences did not reach statistical significance (*p* = 0.49). Regarding age, patients with childhood ALL were stratified into high-risk (<1, ≥10 years) and standard risk (1 to 9.9 years). As shown in Table 2, 54.8% of patients were at standard risk, while only 3 patients (2.1%) were <1 year old and 63 (43.3%) were ≥10 years old. The 5-year OS rate was significantly higher in the standard-risk group (62%) compared to the high-risk group (37%) (*p* < 0.05) (Figure 1A).

An elevated white blood cell count at diagnosis is an adverse prognostic factor in ALL, as it reflects a higher tumor burden and leukemic infiltration of the bone marrow and peripheral tissues. To assess its clinical impact, patients were stratified into two groups: standard risk (<50,000/µL) and high risk (≥50,000/µL). In our cohort, 29 (19.9%) patients had a white blood cell count ≥ 50,000/µL at diagnosis (Table 2). As shown in Figure 1B, the estimated 5-year survival rate was 58% in the standard-risk group, compared with 17.0% in high-risk patients (*p* = 0.0001).

**Figure 2:**
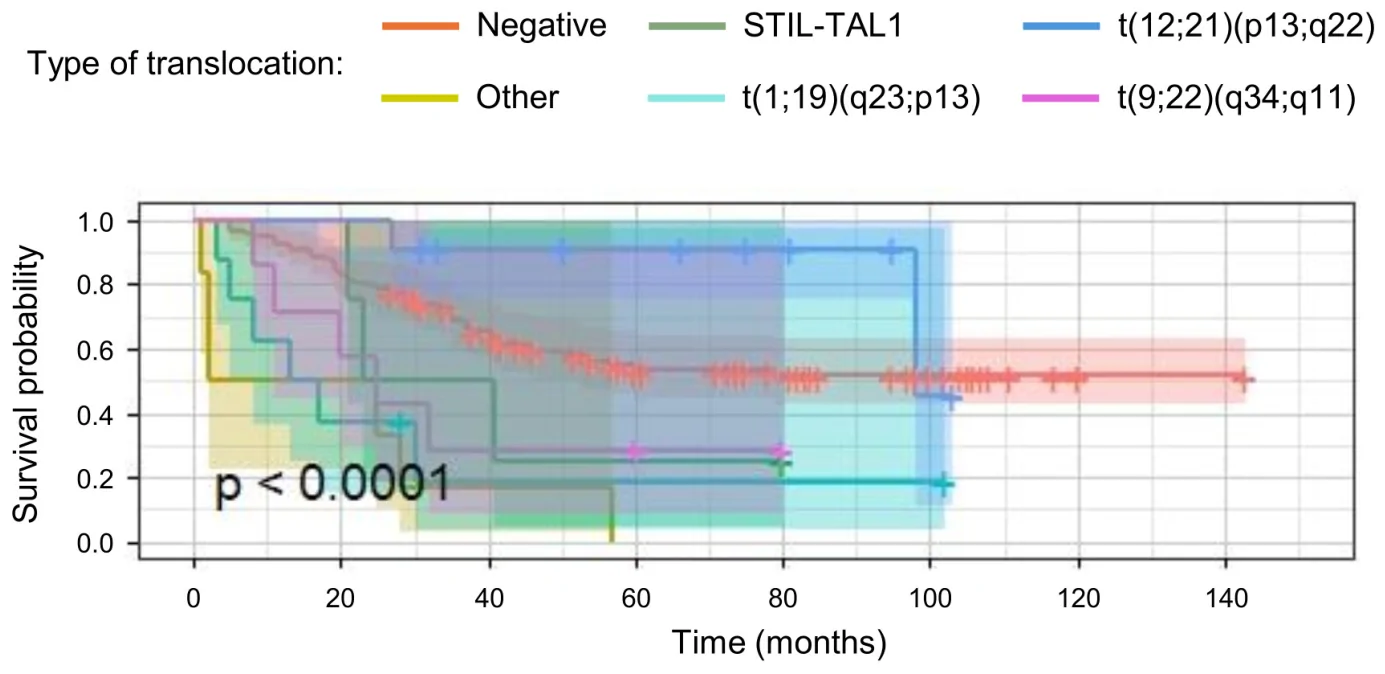
Kaplan–Meier estimates of overall survival by type of translocation. The ETV6::RUNX1 rearrangement was associated with a favorable prognosis, whereas t(9;22), t(1;19), and other less frequent rearrangements, including t(4;11)(q21;q23) and t(8;21)(q22;q22), were linked to poor clinical outcomes (*p* < 0.0001).

Regarding the genetic profile, translocations were identified in 36 of the 146 patients (24.7%). The most frequent were t(12;21)(p13;q22), t(1;19)(q23;p13), t(9;22)(q34;q11), and the STIL-TAL1 fusion transcript, with frequencies of 7.5%, 5.5%, 4.8%, and 2.7%, respectively (Table 2). As shown in Figure 2, patients with the TCF3::PBX1, STIL-TAL and BCR::ABL1 rearrangements were associated with significantly unfavorable survival outcomes (20%, 28.5%, and 30% at 5 years, respectively; *p* < 0.0001). Conversely, patients with the ETV6::RUNX1 rearrangement were linked to improved survival outcomes, reaching a rate of up to 90.9% at 5 years. Other rearrangements detected less frequently, such as t(4;11)(q21;q23), t(8;21)(q22;q22), t(9;11)(p22;q23) and t(11;19)(q23;p13.1), were also associated with a poor outcome; however, none of them were decisive in the stratification of the risk groups.

The total patient cohort was stratified into three risk groups according to modified St. Jude Total XIII and Total XV protocols. Accordingly, 62.3% were classified as high risk, 26.7% as standard risk, and 11.0% as low risk. Because precise MRD monitoring was not systematically available to define early treatment response, this unusually high proportion of high-risk patients was predominantly driven by adverse clinical factors present at the time of diagnosis. Table 3 details the specific clinical and biological drivers for high-risk classification in this cohort.

**Figure 3:**
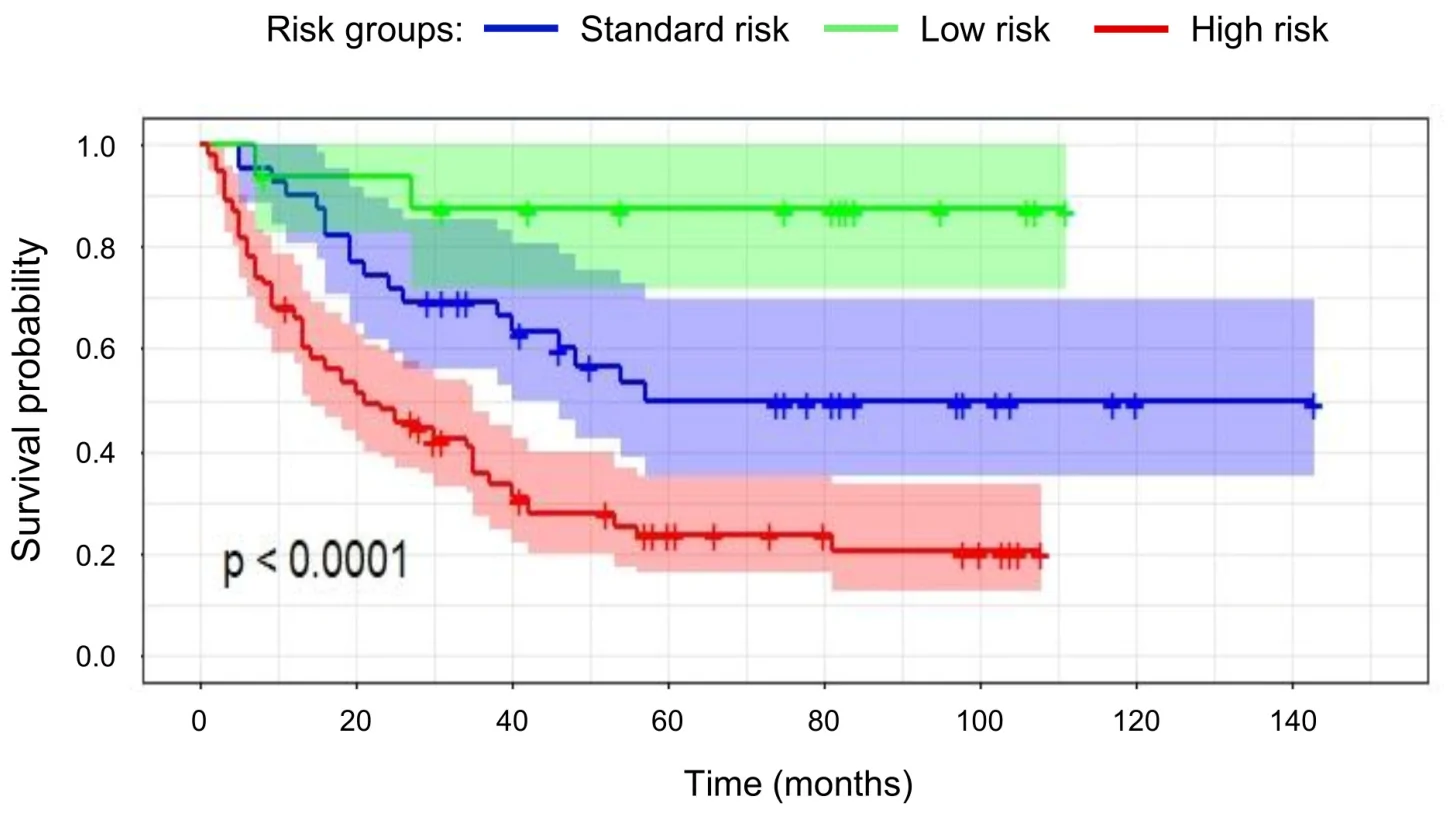
Kaplan–Meier estimates of overall survival stratified by risk groups. Risk classification was determined according to the St. Jude Total XIII and XV protocols, showing outcomes for low-risk (green line), standard-risk (blue line), and high-risk (red line) patients (*p* = 0.0001).

The 5-year survival rate showed significant differences between the groups: 88% for low risk, 50% for standard risk, and 24% for high risk (*p* = 0.0001) (Figure 3).

### 3.3. Relapse and Event-Free Survival

Relapse in ALL represents the main therapeutic challenge and the most adverse prognostic factor. Its prognostic value is intrinsically linked to the timing of the relapse (early relapse, <18 months after diagnosis, being more unfavorable) and to its location, with lower survival rates in bone marrow relapses compared to CNS relapses [10].

Relapse was defined as the recurrence of disease in patients who had achieved complete remission after first-line treatment (blasts < 5% and absence of extramedullary disease) [10]. The overall relapse rate was 45.8%. The main sites of involvement were the CNS (33.5%), bone marrow (18.4%), and combined presentation (7.5%). Regarding timing, very early relapses (<18 months from initial diagnosis) predominated at 30.8% of cases, of which 10.2% affected the bone marrow (Table 2). Early and late relapses accounted for 14.3% and 6.8%, respectively, with cases that presented both early and late relapses.

**Figure 4:**
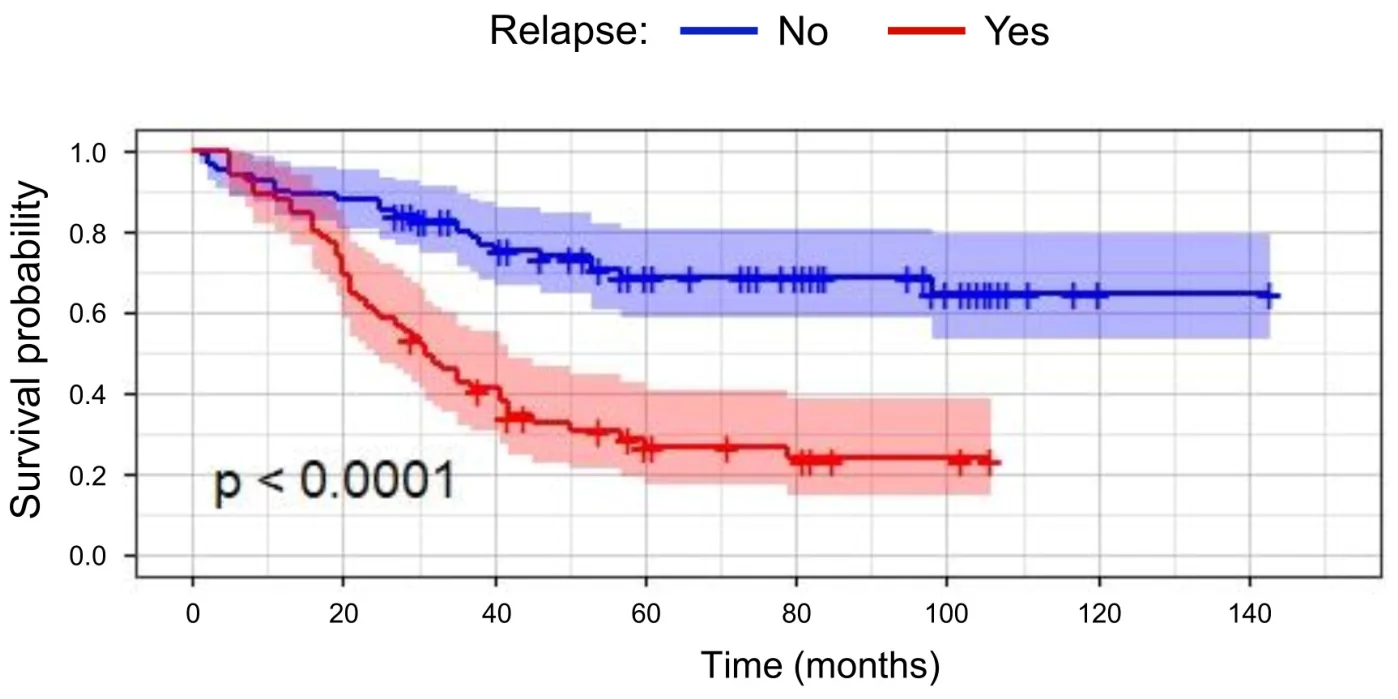
Kaplan–Meier estimates of overall survival stratified by relapse status. Patients who did not experience relapse demonstrated a significantly higher survival probability compared to those with disease recurrence (*p* < 0.0001).

When patients were stratified according to the presence of relapse, statistically significant differences were observed: the group without relapses achieved a 5-year OS of 68%, compared to 29% in those who relapsed (*p* < 0.0001) (Figure 4).

**Figure 5:**
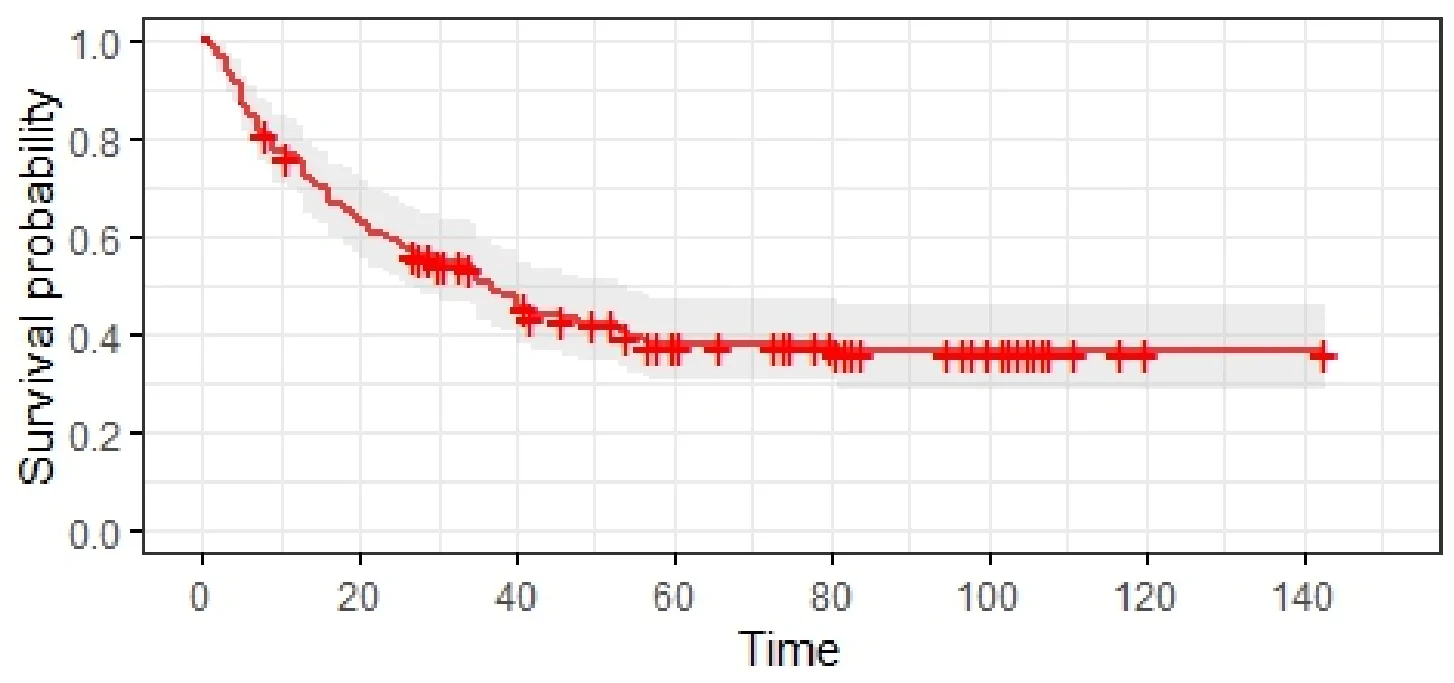
Kaplan–Meier estimates of event-free survival for the entire pediatric B-ALL cohort. Events were defined as the first occurrence of disease relapse or death from any cause. Time is measured in months.

Event-free survival (EFS) was analyzed considering relapse or death as events. During follow-up, 87 of the 146 patients (59.6%) experienced an event. The median EFS was 37 months (95% CI, 26–53 months). The estimated EFS was 59.3% (95% CI, 51.8–67.8%) at 2 years and 37.6% (95% CI, 30.2–47.0%) at 5 years (Figure 5).

**Figure 6:**
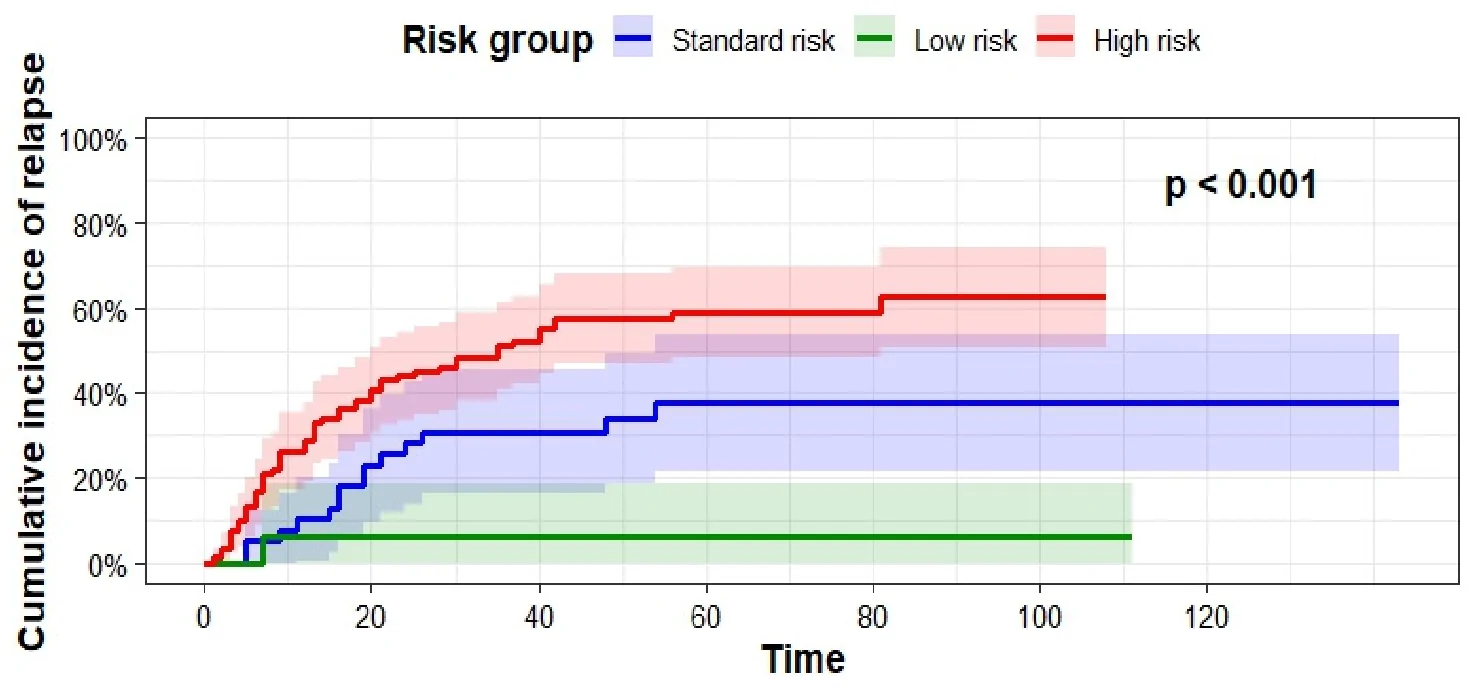
Cumulative incidence of overall relapse in patients with B-ALL stratified by clinical risk group. Estimates were calculated using a competing-risk approach (*p* < 0.001). Time is measured in months.

The cumulative incidence of relapse was estimated using a competing-risk approach considering death without relapse as the competing event. The cumulative incidence of overall relapse was 35.6% (95% CI, 27.8–43.4%) at 2 years and 47.4% (95% CI, 39.0–55.8%) at 5 years. The cumulative incidence of CNS relapse was 26.9% (95% CI, 19.7–34.2%) at 2 years and 37.2% (95% CI, 29.0–45.3%) at 5 years. The 5-year cumulative incidence of relapse showed significant differences between the risk groups (Figure 6).

### 3.4. Impact of Sociodemographic Vulnerabilities and Multivariable Analysis

**Figure 7:**
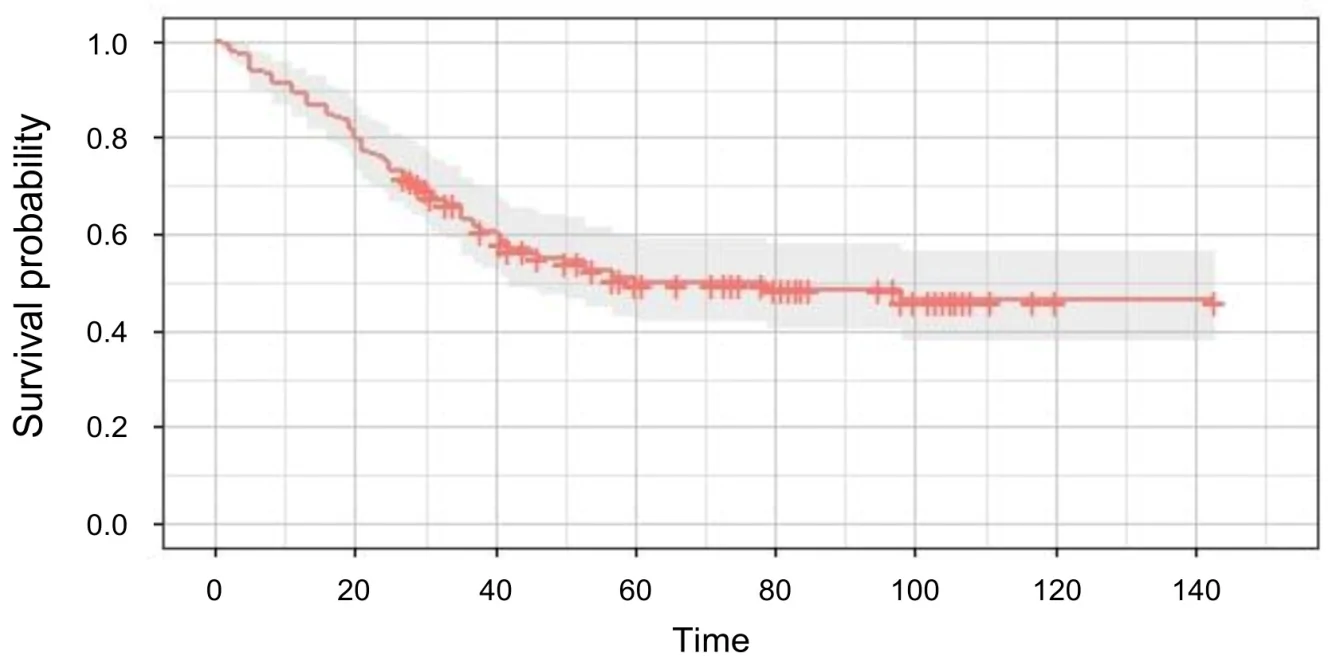
Kaplan–Meier estimates of overall survival rates for the entire pediatric B-ALL cohort. Time is measured in months.

The 5-year overall survival (OS) for the entire cohort was 50% (Figure 7). Beyond established biological parameters, socioeconomic vulnerabilities profoundly impacted these clinical outcomes and follow-up in this cohort. As previously noted, among the 70 deceased patients, the exact terminal medical cause of death was unavailable for 19 patients (27.1%). These cases predominantly reflect patients who were lost to follow-up or passed away in their rural communities outside the hospital setting. This dynamic suggests that the low 5-year OS of 50% is heavily influenced by structural barriers that prevent continuous access to medical care at the end of life, rather than being driven solely by biological factors of the disease.

To further explore healthcare-related factors, additional survival analyses were performed according to documented treatment adherence and indigenous status (Supplementary Table S1). Although indigenous patients showed lower 5-year overall survival (39.9% vs. 51.2%) and event-free survival (24.1% vs. 39.5%) than non-indigenous patients, these differences were not statistically significant (log-rank *p* = 0.287 and *p* = 0.098, respectively). In contrast, patients with documented treatment non-adherence had significantly poorer 5-year overall survival (23.4% vs. 56.1%, log-rank *p* = 0.001) and event-free survival (12.8% vs. 43.5%, log-rank *p* = 0.007) compared with adherent patients.

While treatment non-adherence could not be included in the multivariable Cox regression due to temporal bias constraints, descriptive analysis revealed its profound intersection with socioeconomic vulnerabilities. Notably, documented treatment non-adherence was drastically more prevalent among indigenous patients. Our data shows that 55.5% (15 out of 27) of patients with indigenous status experienced severe treatment interruptions, compared to only 10.1% (12 out of 119) of non-indigenous patients.

To identify independent prognostic factors and adjust for overlapping clinical and sociodemographic variables, multivariable Cox proportional hazards regression analyses were performed for overall survival and relapse (Table 4). High clinical risk remained the only independent predictor of overall mortality (HR = 4.15, 95% CI 1.72–10.03; *p* = 0.001). In contrast, male sex (HR = 1.79, 95% CI 1.05–3.10; *p* = 0.033), high leukocyte count (HR = 2.22, 95% CI 1.20–4.10; *p* = 0.011), and indigenous status (HR = 1.89, 95% CI 1.00–3.50; *p* = 0.049) were independently associated with an increased hazard of relapse, with the latter serving as a critical proxy for these structural and adherence-related barriers. No significant associations were observed for age, translocation status, or geographic region after multivariable adjustment (Table 4).

## 4. Discussion

Multivariate analysis identified several factors associated with survival outcomes. Age, white blood cell count, presence of translocations, and relapses were significantly associated with OS. Patients aged 1 to 9.9 years predominated, representing 54.8%. The <1 and ≥10 age groups were significantly associated with a higher risk of mortality (38% at 5 years) (*p* = 0.016) (Figure 1), which is consistent with previous studies [11,12]. Furthermore, 19.9% of patients had a white blood cell count ≥ 50,000/μL. This value was significantly associated with a higher risk of mortality, resulting in an overall 5-year survival rate of 15% (*p* = 0.045). The high prevalence of hyperleukocytosis (≥50,000/µL) observed in our cohort is closely linked to delayed diagnosis. In our region, severe socioeconomic constraints, geographical isolation, and linguistic barriers frequently lead to late medical presentations, allowing unabated disease progression and higher tumor burdens at admission. Consequently, these social determinants directly skew baseline risk profiles toward higher clinical risk upon arrival.

Recurring chromosomal rearrangements are a hallmark of ALL and can be acquired during leukemogenesis or disease progression [13] and are related to the risk of relapse [7]. The frequency and prognostic relevance of each type of alteration vary considerably between leukemia subtypes [13]. Detecting these alterations is clinically important for risk stratification, monitoring of MRD, and in some cases, for implementing targeted therapies [14].

In this study, the most frequent chromosomal rearrangement was ETV6-RUNX1, detected in 11 patients (7.5%). The t(12;21)(p13;q22) translocation is a well-established cytogenetic alteration in B-cell ALL linked to a favorable prognosis and excellent clinical outcomes. The frequency of this translocation in the literature is variable: it was reported as 18.2% and 23% by Qiu KY et al. [15] and Lee JW et al. [16], respectively. In line with these findings, in our cohort, the t(12;21)(p13;q22) translocation was also associated with a favorable prognosis, showing a 5-year survival rate of 90.9% (*p* = 0.0074) (Figure 3). This result is consistent with that reported by Lee JW et al. [16], who documented a 10-year survival rate of 79.5% ± 4.4% for these patients, *p* = 0.033. On the other hand, the t(9;22)(q34;q11.2) translocation, which encodes the Philadelphia chromosome and is associated with an adverse prognosis, was detected in 4.8% of our patient cohort. The frequency of this translocation is markedly higher than that reported in a retrospective ALL cohort study by Campbell M et al. [17], who reported a frequency of only 2.0%. It is worth noting that our patients received tyrosine kinase inhibitors (imatinib) in addition to standard chemotherapy, improving survival compared to those with other translocations (Figure 3). The low prevalence of the ETV6-RUNX1 gene fusion and the high frequency of BCR::ABL1 in our pediatric cohort could be factors that significantly influence the unfavorable prognosis and low overall survival observed in our patients.

The t(1;19)(q23;p13) translocation, which results in the TCF3-PBX1 fusion, was the second most frequent alteration in this study (5.5% of cases) (Table 2). This finding is consistent with the literature, specifically with Lee JW et al. [16], who reported a frequency of 7.7% and, as in this study, identified it as the second most common alteration, as well as with Iacobucci I et al. [14], who reported a prevalence of 5–6%. In our cohort, this translocation was associated with a poor prognosis, evidenced by the fact that 75% of patients positive for this genetic abnormality experienced early relapse of the CNS, with a negative impact on overall survival (40% at 5 years). This finding is relevant because, although some studies have mitigated its impact with intensive therapy, it coincides with research suggesting that t(1;19)(q23;p13) may still be an independent risk factor for CNS relapse [14].

Other chromosomal rearrangements associated with poor prognosis and low response rate to standard chemotherapy detected in our patient cohort included t(4;11)(q21;q23), which produces the KMT2A::AFF1 fusion gene, detected in two infants, aged 1 year and 9 months, respectively. The latter died due to tumor lysis syndrome/renal failure after treatment was initiated.

The 5-year overall survival (OS) identified in this study was 50%. This value is significantly lower than the 67.5% (±1.6%) reported by Moreira DC et al. [18] for B-ALL in Mexico. The disparity is even more pronounced when comparing these results with those of high-income countries, where OS can reach 94%, as is the case in the United States [19].

The event-free survival rate was similarly low, reflecting the substantial burden of relapse and death observed during follow-up. Since EFS captures both mortality and disease recurrence, it may provide a more comprehensive assessment of treatment effectiveness than OS alone. In our cohort, the estimated 5-year EFS was 37.6%. This outcome stands in stark contrast to the results reported by Pui et al. in the prospective St. Jude Total Therapy XV trial, which achieved a 5-year EFS of 87.3% [20]. The profound disparity in EFS, despite our institution’s use of a modified version of the same Total XV protocol, underscores the critical impact of structural and socioeconomic barriers. Specifically, the St. Jude study utilized sequential, highly sensitive MRD monitoring to dynamically tailor treatment intensity and successfully mitigate relapse risk—a strategy that was not systematically feasible in our resource-limited setting.

Regarding disease recurrence, the cumulative incidence of overall relapse in our cohort reached 47.4% at 5 years, with 45.8% of patients experiencing at least one relapse during the first 5 years (11 of these patients presented with combined relapse). This number considerably exceeds the 12.5% reported by Rheingold et al. [10] and the cumulative risk of relapse at 10 years of 7.2% for provisional low-risk and 15.3% for standard-risk patients reported in the St. Jude Total Therapy XV study [20]. Considering the site of relapse, 27 (18.4%) patients in our cohort experienced relapse in the bone marrow (Table 2). Other sites included the CNS in 49 patients (33.5%), with testicular relapse being less common. A total of 70.1% experienced a single episode, while 29.8% experienced ≥2 relapses. Regarding timing, relapses occurred predominantly in very early (10.2% bone marrow and 20.5% central nervous system) stages, which coincides with what was reported by Chen X, Yu J [21], who revealed predominance in the very early stage (42.5%). The overall 5-year survival rate after relapse was 28% (Figure 5), which contrasts with that reported by Rheingold et al. [10] and Chen X, Yu J [21], who reported 5-year post-relapse OS rates for B-ALL of 52.5 ± 1.3% and 47.2%, respectively. These findings reinforce that relapse, particularly involving the central nervous system, remains a major contributor to treatment failure despite protocol-based therapy.

The high incidence of relapses and the low survival rate observed in our cohort reflect not only the influence of biological prognostic factors but also social determinants and cultural barriers inherent to our study population. A crucial aspect is that most patients belong to groups with limited economic resources. As revealed by our audit, 27 patients (18.4%) had officially documented non-adherence to treatment. Importantly, these incidents of non-adherence—characterized by intermittent delays or interruptions in chemotherapy—were predominantly driven by severe socioeconomic constraints, language barriers, and the chronic inability to secure blood donors, rather than by direct medical toxicity. This economic vulnerability is compounded by the generally low level of education and the significant proportion of patients belonging to indigenous groups (Nahua, Mixtec, Tlapaneco, and Amuzgo). In our cohort, 18.5% of patients belong to an indigenous group with little or no Spanish. The language barrier creates serious difficulties on two critical fronts: (1) obtaining timely blood and blood product donors, and (2) a thorough understanding of the disease and, fundamentally, the importance of strict adherence to the treatment protocol. This treatment non-adherence frequently led to readmissions in serious clinical conditions such as tumor lysis syndrome, abdominal sepsis, and early relapse.

Furthermore, the successful implementation of highly intensive regimens like the St. Jude protocols is intrinsically tied to the availability of high-quality supportive care, an area where our institution faces profound structural limitations. Our center operates without a pediatric intensive care unit (PICU), which heavily restricts our capacity to safely manage critical chemotherapy-induced toxicities or septic shock. This infrastructural deficit is compounded by the aforementioned social vulnerabilities; because patients lack support networks in the city, the chronic difficulty in securing mandatory blood donors leads to prolonged periods of severe anemia and thrombocytopenia during myelosuppression. Consequently, these critical deficiencies in supportive care infrastructure significantly increase the risk of fatal hemorrhagic and infectious events, contributing to the overall inferior survival outcomes observed in this cohort.

Similarly, while hematopoietic stem cell transplantation (HSCT) is the standard of care for high-risk and relapsed ALL in high-income countries, this therapeutic modality was not available for any patient in our cohort. Safely performing HSCT requires an advanced blood bank with the capacity to immediately provide irradiated and filtered blood products on demand, a logistical capacity our center currently lacks. The inability to offer HSCT as a consolidation strategy profoundly limits our therapeutic arsenal and contributes to the high post-relapse mortality observed.

To statistically delineate the interplay between these systemic vulnerabilities and intrinsic disease biology, our multivariable Cox regression analyses evaluated the divergent impact of clinical and sociodemographic factors. High clinical risk emerged as the sole independent predictor of overall mortality (HR = 4.15, *p* = 0.001), reinforcing the foundational prognostic value of clinical risk stratification in pediatric ALL [22]. Conversely, distinct variables—specifically male sex (HR = 1.79, *p* = 0.033), high leukocyte count (HR = 2.22, *p* = 0.011), and indigenous status (HR = 1.89, *p* = 0.049)—were independently associated with an increased hazard of relapse. Although initial leukocyte count showed a borderline association with overall survival, its prominent role as an independent driver of disease recurrence aligns with previous reports identifying hyperleukocytosis as a major adverse prognostic factor in pediatric leukemia [23]. It is essential to emphasize that indigenous status does not imply a biological or genetic predisposition to relapse among indigenous populations. Rather, indigenous status serves as a powerful proxy for the severe structural inequities described above. The stark disparity in treatment non-adherence—affecting 55.5% of indigenous patients compared to only 10.1% of their non-indigenous peers—highlights a systemic failure in healthcare delivery. Geographic and socioeconomic disparities have consistently been shown to shape treatment adherence and survival trajectories in oncological care [24]. These findings indicate that while overall mortality is dictated by the biology of the disease, the initial failure of therapy in our setting is largely driven by treatment non-adherence. For instance, a recent Children’s Oncology Group (COG) study demonstrated that the association of race and ethnicity with worse post-relapse survival is largely accounted for by adverse disease-related factors present at the time of relapse, such as early recurrence and initial hyperleukocytosis [25]. However, even after adjusting for these clinical parameters, ethnicity and socioeconomic vulnerabilities—such as language barriers and lower household income—persist as independent predictors of inferior outcomes [25,26]. This underscores that while clinical risk defines the biological salvageability of a relapse, profound socioeconomic constraints and communication barriers, such as those prevalent in our indigenous and rural cohort, critically compromise the ultimate survival of these patients.

Our study has several limitations. First, due to infrastructural constraints and frequent reagent shortages, MRD monitoring at the end of induction was not consistently performed across all patients at standardized time points; in some instances, testing was only requested upon clinical suspicion of relapse. This diagnostic limitation represents a critical challenge in our real-world setting. As a result, the atypically high proportion of high-risk patients (62.3%) was defined almost entirely by baseline clinical features rather than documented poor early response to therapy. This reality reinforces our observation that delayed medical attention allows the disease to progress to advanced stages before admission, forcing pediatric oncologists to classify the vast majority of patients as high-risk based on severe presentation features like hyperleukocytosis and primary CNS infiltration. This contrasts starkly with modern prospective trials—such as the St. Jude Total XV protocol—where systematic MRD monitoring allows for precise, dynamic risk reclassification and targeted treatment modification after induction therapy [20]. Second, although treatment non-adherence represents a critical barrier in our population, it could not be appropriately incorporated as a time-dependent covariate in our multivariable Cox regression models because the exact timing of treatment interruptions was not systematically recorded. Including it as a fixed baseline predictor would introduce immortal time bias; therefore, its impact is addressed through descriptive analysis rather than multivariable adjustment. Furthermore, detailed individual-level data on medication availability, family income, insurance status, and other healthcare delivery factors were unavailable due to the retrospective design. Consequently, their contribution to mortality and relapse could not be directly quantified. Finally, it was not possible to accurately determine treatment-related mortality (TRM) for the entire cohort. While disease progression was the primary cause of death for most patients, the exact terminal cause remained unknown for a significant subset (27.1%). This missing data was mainly due to loss to follow-up and patient deaths in remote rural communities, which may have led to an underestimation of the true TRM. Future research will be essential to evaluate whether the implementation of newer protocols, along with improved access to consistent MRD monitoring, leads to a significant improvement in clinical outcomes.

## 5. Conclusions

In conclusion, this retrospective cohort study of pediatric B-ALL in Southern Mexico revealed a 5-year OS of 50% and an EFS of 37.6%, rates markedly lower than those reported in high-income countries. While traditional clinical features—such as age, high white blood cell count, and high-risk cytogenetic alterations—remain important prognostic factors, our findings demonstrate that socioeconomic vulnerabilities are critical, independent drivers of therapeutic failure. Specifically, multivariable Cox regression analysis identified indigenous status as a significant independent predictor of relapse. Rather than reflecting a biological predisposition, this association serves as a proxy for severe structural inequities, evidenced by the high rates of treatment non-adherence observed among this vulnerable population. Ultimately, closing this significant survival gap in resource-limited settings requires moving beyond purely biological risk stratification. It is imperative to integrate targeted social support programs and equitable healthcare delivery strategies designed to overcome socioeconomic barriers, prevent treatment non-adherence, and improve long-term clinical outcomes.

## Figures and Tables

**Table 1:** Criteria for risk stratification of patients with childhood ALL according to protocols Total XIII and Total XV.

Low Risk	Standard Risk	High Risk
Pre-B Age 1–9.9 years ETV6-RUNX1 translocation Total leukocytes < 50,000 Do not present: CNS-3, testicular ALL, t(9;22) BCR/ABL, t(1,19) E2A/PBX, MLL(11q23), MRD+, >1% blasts on day 26.	Same as for low risk, except for the absence of trisomies 4, 10 and 17 or ETV6-RUNX1	t(9;22) BCR/ABL Induction failure or >1% blasts in bone marrow. MRD+ Bone marrow > 0.1% blasts on follow-up (14 weeks post-induction) Initial CNS or testicular involvement

CNS, central nervous system; CNS-3, >5 leukocytes/μL with evidence of blasts on cytocentrifuge examination or cranial nerve involvement; ALL, acute lymphoblastic leukemia; MRD, minimal residual disease.

**Table 2:** Clinical and demographic characteristics of patients with childhood ALL.

Characteristics	Overall *N* = 146
Age (years) at diagnosis	
median (IQR)	8 (4–13)
Age groups	
1–9.9	80 (54.8%)
≥10	63 (43.3%)
<1	3 (2.1%)
Sex	
Male	74 (50.7%)
Female	72 (49.3%)
Status	
Alive	76 (52.1%)
Dead	70 (47.9%)
White blood cell count	
WBC < 50,000	117 (80.1%)
WBC ≥ 50,000	29 (19.9%)
Translocation	
Positive	36 (24.7%)
Negative	110 (75.3%)
Most frequent translocations	
t(12;21)(p13;q22)	11 (7.5%)
t(1;19)(q23;p13)	8 (5.5%)
t(9;22)(q34;q11)	7 (4.8%)
STIL-TAL1	4 (2.7%)
Other	6 (4.1%)
Negative	110 (75.3%)
Risk group	
High	91 (62.3%)
Standard	39 (26.7%)
Low	16 (11.0%)
Time of relapse	
Very early	45 (30.8%)
Early	21 (14.3%)
Late	10 (6.8%)
CNS relapse	
Negative	97 (66.4%)
Positive	49 (35.5%)
Bone marrow relapse	
Negative	119 (81.6)
Positive	27 (18.4%)

*N* = Overall number; IQR = Interquartile Range; WBC = White blood cell count; CNS = Central nervous system.

**Table 3:** Clinical and biological factors define high-risk classification in the cohort (*N* = 91).

High-Risk Determinant Factor	Number of Patients (*n*)	Percentage of High-Risk Group
Demographic and clinical factors at diagnosis		
High-risk age (<1 year or ≥10 years)	65	71.4%
Hyperleukocytosis (WBC ≥ 50,000/µL)	28	30.8%
Primary central nervous system (CNS) infiltration	10	10.9%
Primary infiltration of another site (optic nerve or testis)	6	6.5%
Prior steroid use	3	3.2%
Genetic factors (Cytogenetics)		
Presence of t(9;22) BCR/ABL (Philadelphia chromosome)	7	7.7%
Down syndrome	1	1.1%
Early treatment response		
Documented induction failure Refractory to therapy	16	17.5%
Positive minimal residual disease (MRD)	Not systematically available	N/A

Note: Percentages do not sum to 100% as a single patient may present with multiple overlapping high-risk factors at diagnosis. N/A = Not Available.

**Table 4:** Multivariable Cox proportional hazards regression analyses identifying independent predictors of overall survival and relapse.

**Overall survival**				
**Variable**	**Category (vs. Reference)**	**HR**	**95% CI**	***p*** **-Value**
Sex	Male vs. Female	1.45	0.88–2.39	0.149
Age group	0–0.9 vs. 1–9 years	1.48	0.38–5.70	0.568
	10–18 vs. 1–9 years	0.75	0.39–1.43	0.386
Leukocyte count	High risk vs. standard	**1.81**	**0.98–3.33**	**0.056**
Translocation	STIL-TAL1 vs. negative	1.24	0.38–4.12	0.721
	t(1;19) vs. negative	2.24	0.89–5.65	0.088
	t(12;21) vs. negative	0.47	0.11–1.99	0.304
	t(9;22) vs. negative	1.02	0.38–2.80	0.962
Clinical risk	High vs. standard	**4.15**	**1.72–10.03**	**0.001**
	Low vs. standard	0.53	0.11–2.44	0.414
Region	Center vs. Acapulco	1.16	0.54–2.46	0.708
	Coast vs. Acapulco	**0.47**	**0.22–1.01**	**0.054**
	Other vs. Acapulco	0.73	0.37–1.45	0.362
Indigenous status	Yes vs. No	1.7	0.92–3.17	0.093
**Relapse**				
**Variable**	**Category (vs. reference)**	**HR**	**95% CI**	***p*** **-value**
Sex	Male vs. Female	**1.79**	**1.05–3.10**	**0.033**
Age group	0–0.9 vs. 1–9 years	1.71	0.33–8.90	0.524
	10–18 vs. 1–9 years	1.55	0.73–3.33	0.252
Leukocyte count	High risk vs. standard	**2.22**	**1.20–4.10**	**0.011**
Translocation	STIL-TAL1 vs. negative	1.30	0.38–4.40	0.668
	t(1;19) vs. negative	1.86	0.68–5.14	0.228
	t(12;21) vs. negative	0.16	0.02–1.20	0.076
	t(9;22) vs. negative	1.87	0.65–5.41	0.248
Clinical risk	High vs. standard	1.75	0.69–4.40	0.238
	Low vs. standard	0.15	0.02–1.20	0.070
Region	Center vs. Acapulco	1.63	0.74–3.60	0.223
	Coast vs. Acapulco	0.70	0.32–1.50	0.370
	Other vs. Acapulco	0.84	0.40–1.80	0.647
Indigenous status	Yes vs. No	1.89	1.00–3.50	0.049

Significant values are denoted in bold.

## Data Availability

The data supporting reported results can be found in the Supplementary Material, specifically provided as Supplementary Files S3 and S4.

## Supplementary Materials

The following supporting information can be downloaded at: https://www.mdpi.com/article/10.3390/jcm15176758/s1. Supplementary File S1: Modified St. Jude Total XIII; Supplementary File S2: Modified St. Jude Total XV; Supplementary Table S1: Five-year overall survival and event-free survival according to indigenous status and treatment adherence; Supplementary File S3: Clinical and demographic dataset of the cohort; Supplementary File S4: Data Dictionary of the Cohort.

## Abbreviations

B-ALL = B-Cell Acute Lymphoblastic Leukemia, OS = Overall Survival, WBC = White blood Cell, MRD = Minimal Residual Disease, IECAN = State Cancer Institute, RT-PCR = Reverse Transcription Polymerase Chain Reaction, CNS = Central Nervous System, HR = R Hazard Ratio, TRM = Treatment-related mortality, EFS = Event-free survival, COG = Children’s Oncology Group, HSCT = hematopoietic stem cell transplantation, PICU = pediatric intensive care unit.
