# Supplementary material for: Clinical Outcome and Prognostic Factors in Childhood Acute Lymphoblastic Leukemia—A Retrospective Cohort Study of Patients from Southern Mexico
Source: J Clin Med. 2026 Aug 31;15(17):6758. doi: 10.3390/jcm15176758 (PMC13566665; doi:10.3390/jcm15176758)
Supplement: Supplementary file 1 [file jcm-15-06758-s001.zip › Supplementary Table S1.pdf]

Supplementary Table S1

Five-year overall survival and event-free survival according to healthcare-related contextual factors

Variable	Group	5-year OS (95% CI)	p	5-year EFS (95% CI)	p
Indigenous status	Non-indigenous	51.2 (42.6–61.7)	0.287	39.5 (31.1–50.0)	0.098
	Indigenous	39.9 (24.3–65.6)		24.1 (11.6–50.1)	
Treatment adherence	Adherent	56.1 (47.4–66.3)	0.001	43.5 (35.0–53.9)	0.007
	Non-adherent	23.4 (11.5–47.8)		12.8 (4.5–36.5)	
