# Supplementary material for: Clinical Outcome and Prognostic Factors in Childhood Acute Lymphoblastic Leukemia—A Retrospective Cohort Study of Patients from Southern Mexico
Source: J Clin Med. 2026 Aug 31;15(17):6758. doi: 10.3390/jcm15176758 (PMC13566665; doi:10.3390/jcm15176758)
Supplement: Supplementary file 1 [file jcm-15-06758-s001.zip › Supplementary_File_S1.pdf]

TOTAL TECHNICAL PROTOCOL XIII MODIFIED

The risk criteria:

Low Risk/Standard Risk

1. Leukocyte count less than 50 × 109/L, 1 to 9 years old.
2. DNA index from 1.16 to 1.60.
3. CNS-1 status (leukocytes < 5/µL without leukemic blasts in the cerebrospinal fluid (CSF).
4. Good early response to treatment) morphological examination of the bone marrow < 5%) on day 15 of the induction phase in the absence of T-cell leukemia, testicular infiltration, and t(1:19), t(4; 11), and t(9:22) of the pH chromosome and rearrangement of the MLL gene

High risk

1. T-cell leukemia.
2. Testicular infiltration
3. T(1:19)
4. T(4; 11)
5. T(9:22) of the pH chromosome
6. MLL genetic rearrangement

TREATMENT REGIMEN FOR USUAL-RISK/LOW-RISK ACUTE LYMPHOBLASTIC LEUKEMIA (ALL)

Total chemotherapy regimen is started with an induction schema to remission:

· Days 7 to 0: prednisone, 60 mg/m2sc/day, divided into three doses. Maximum dose 60 mg.
· Days 0, 7, 14, 21, 28: vincristine, 2 mg/m2sc/day. Maximum dose 2 mg.
· Day 0 and day 14: daunorubicin, 30 mg/m2sc/day.
· Días 5, 8, 12, 15, 19, 22: L-asparaginase, 10 000 UI/m2sc/day, IM.
· Days 0 to 28: prednisone, 60 mg/m2sc/day, VO; from day 29 onwards, reduction begins.
· Days 0, 14, 28:

Age	< year	1 to 2	2 to 3	3 to 8	>8
Methotrexate (mg)	6	8	10	12	15
Hydrocortisone (mg)	6	8	10	12	15

On day 28, bone marrow aspirate (AMO) was taken again to evaluate whether complete remission was achieved: this is considered if there are less than 5% blasts and the rest of the cellularity is restored.

Once complete remission is confirmed, start consolidation according to the following scheme:
· Consolidation: first cycle, methotrexate (start if neutrophils ≥ 1 000/mm3 are present).
· Day 35: Vincristine, 2 mg/m2sc/day.
· Day 36: methotrexate, 2 g/m2sc, one-tenth to be administered in rapid dose (bolus) and remaining doses by infusion in 24 hours.
Intrathecal chemotherapy 1 h after IV methotrexate bolus, dose according to age:

**Age**	**< 1 year**	**1 to 2**	**2 to 3**	**3 to 8**	**> 8**
Methotrexate (mg)	6	8	10	12	15
Hydrocortisone (mg)	6	B	10	12	15
Cytarabine (mg)	12	16	20	24	30

· Day 37: Initiate rescue with leucovorin 15 mg/m2sc/day, 12 h after completion of methotrexate infusion (10 doses total).

Rest for five to seven days after concluding the rescue with leucovorin; continue consolidation with (consider neutrophils ≥ 1 000/mm3) (CAP-VAP):

· Day 44: Cyclophosphamide, 1 000 mg/m2sc/day.
· Days 45, 46, 47, 48 and 52, 53, 54, 55: ara C, 80mg/m2sc/day IV.

· Days 45 to 59: 6-mercaptopurine, 50 mg/m2sc/day, PO.
· Day 51: Vincristine, 2 mg/m2sc/day (maximum dose 2 mg).

Rest for 10 to 14 days, and with a neutrophil count ≥ 1 000/mm3, continue the second cycle of methotrexate, which is administered in the same way as the first cycle.

· Day 67: Vincristine, 2 mg/m2sc/day.
· Day 68: methotrexate, 2 g/m2sc/in 24 h, one-tenth will be bolus, and the remaining doses will be given by infusion within 24 hours.

· Metotrexato, 15 mg/m2sc/día, IT.
· Hydrocortisone, 15 mg/m2sc/day, IT.
· Ara-C. 30 mg/m2sc/día, IT.

· Day 69: leucovorin, 15 mg/m2sc/day for 10 doses.

Rest for 7 to 10 days, and with a neutrophil count ≥ 1 000/mm3, continue:

· Day 76: Cyclophosphamide, 1 000 mg/m2sc/CFM 300 Xd
· Days 77. 78, 79, 80 and 84, 85, 86, 87: ara C, 80mg/m2sc/day.
· Days 77 to 90: 6-mercaptopurine, 50 mg/m2sc/day, PO.
· Day 83: Vincristine, 2 mg/m2sc/day (maximum dose 2 mg).

Rest for 10 to 14 days, and with a neutrophil count ≥ 1 000/mm3, continue the third cycle of methotrexate equal to the first:
· Day 93: Vincristine, 2 mg/m2sc/day.
· Day 94: methotrexate, 2 g/m2sc/in 24 h, one-tenth is given as a bolus and the remaining dose by infusion within 24 hours.

· Metotrexato, 15 mg/m2sc/day, IT.
· Hydrocortisone, 15 mg/m2sc/day, IT.
· Ara-C, 30 mg/m2sc/día, IT.
· Day 95: leucovorin, 15 mg/m2sc/day.

From this moment on, intrathecal chemotherapy (IT) is applied at the doses already mentioned, every month for six months.

Rest for 10 to 14 days, and with a neutrophil count ≥ 1 000/mm3, continue (TAAP).

· Day 102: 6-mercaptopurine, 50 mg/m2sc/day, PO.
· Days 102 to 106: ara C, 100 to 120 mg/m2sc/day
· Day 106: doxorubicin, 30 mg/m2sc/day.
· Days 102 to 106: prednisone, 60 mg/m2sc/day.

Rest for 10 to 14 days, and with a neutrophil count ≥ 1 000/mm3, continue (TAAP).

· Days 120 to 176: 6-mercaptopurine, 50 mg/m2sc/day, daily fasting PO.
· 8 weeks: methotrexate 20 mg/m2sc/week, PO divided into two days a week and two doses per day (evening and night).

With neutrophil count ≥ 1 000/mm3
Continue:
· Days 177, 184, 191, 198: vincristine, 2 mg/m2sc/day (maximum dose 2 mg).
· Days 177, 191: daunorubicin, 30 mg/m2sc/day.
· Days 177 to 198: prednisone, 60 mg/m2sc/day, VO.

(From day 215 onwards, reduction begins)
· Días 182, 185, 189, 192, 196, 199: L-asparaginase, 10 000UI/m2sc/day, IM.
Rest for seven days; continue intensification with (consider neutrophils ≥ 1 000/mm3)
(CAP-VAP):

· Day 207: Cyclophosphamide, 1 000 mg/m2sc/day.
· Days 208, 209, 210, 211 and 215, 216. 217. 218: ara C, 80 mg/m2sc/day.
· Days 208 to 222: 6-mercaptopurine, 50 mg/m2sc/day, PO.
· Day 214: Vincristine, 2 mg/m2sc/day (maximum dose 2 mg).

Rest for 10 to 14 days, and with neutrophil count ≥1 000/mm3, continue (TAAP).
· Day 236: 6-mercaptopurine, 50 mg/m2sc/day, PO.
· Days 236 to 240: now C. 100 to 120 mg/m2sc/day
· Day 240: doxorubicin, 30 mg/m2sc/day.
. Days 236 to 240: prednisone, 60 mg/m2sc/day.
· Days 247 to 331: 6-mercaptopurine, 50 mg/m2sc/day, vo.

12 weeks: methotrexate, 20 mg/m2sc/week, PO, divided into two days a week and two doses per day (evening and evening).

With neutrophil count ≥1 000/mm3,
Continue:
· Days 332, 339. 346, 355: vincristine, 2 mg/m2sc/day (maximum dose 2 mg).
· Days 332 to 355: prednisone, 60 mg/m2sc/day, PO.

From day 355 onwards, the reduction begins

· Días 337, 340, 344, 347, 353, 356: L-asparaginase, 10 000UI/m2sc/day, IM.

From day 380 (or sixth monthly intrathecal dose), continue with intrathecal triple drug (ITT) every two months for six doses; then, continue with TIT every three months until 900 days of continuous complete remission (30 m of RCC) are completed.

TREATMENT SCHEDULE FOR HIGH-RISK ALL

Induction	Doses	Route of administration
· Vincristine	1.5 mg/m²/week/4 weeks days 1,8,15, 22 (Dosage max. 1.5 mg)	IV
· Prednisone	window day 4 a 40 mg/m2/day/28 days (Dosage máx. 60 mg/day)	Oral
· Doxorubicin	25 mg/m2 days 1 and 8	Ora
· L-asparaginase	10 000 Ul/m2, days 2,4,6,8,10,12; add days 15, 17 and 19 if there are > 5% blasts (MO) on day 15	IM
· Etoposide	300 mg/m2 days 22,25 y 29	Infusion in 1 h
· Cytosine arabinoside	300 mg/m2 days 22,25 y 29	Infusion in 1 h
· Triple Drug intrathecal	dose by age (see Table 4-3) on days 5 and 26, if the CSF is + blasts (CNS 3) days 12 and 19 are added. In remission all will receive 1/week/4 weeks, then every 4 weeks/1 year	IT
Radiotherapy	Skull with CSF-CNS 3 only	24 Gy in 16 fractions from week 56 to 59

**Consolidation**		
(start of weeks after induction)		
· Methotrexate	2 g/m2 with leucovorin rescue at 30 mg/m2/dose, IV, every 3 h a 42 h after starting methotrexate, until levels are reached serum > MTX of 0.03 uMol/L, on days 47 and 54	Infusion in 24 h
6-mercaptopurine	75 mg/m2/day, from day 47 to day 61	VO at night

**Treatment post-remission**		
(Duration 120 weeks)		
· Etoposide	300 mg/m2 week 1	Infusion in 1 h
Cyclophosphamide	300 mg/m2 week 1	Infusion in 1 h
· Methotrexate	40 mg/m2 week 2	IM
· 6-mercaptopurine	75 mg/m2 diario/7 days, week 2	VO at night
Methotrexate	40 mg/m2 week 3	IM
· Cytosine arabinoside	300 mg/m2 week 3	Infusion in 1 h
· Vincristine	1.5 mg/m2 week 4	IV
· Dexamethasone	8 mg/m2 diary/ 7 days, week 4	VO 3 veces/dia
· Etoposide	300 mg/m2 week 5	Infusion in 1 h
· Cyclophosphamide	300 mg/m2 week 5	Infusion in 1 h
· Methotrexate	2 g/m2 with leucovorin rescue at 30 mg/m2/dose. IV, every 3 h at 42 h after starting methotrexate, week 6	Infusion in 24 h
· 6-mercaptopurine	75 mg/m2/day/ 7 days, week 6	VO at night
· Etoposide	300 mg/m2 week 7	Infusion in 1 h
· Cytosine arabinoside	300 mg/m2 week 7	Infusion in 1 h
· Vincristine	1.5 mg/m2 week 8	IV
· Dexamethasone	8 mg/m2 Daily/ 7 days, week 8	VO 3 times/day
Weekly chemotherapy with paired drugs is repeated until 120 weeks are completed		

**Re-induction treatment**		
The same agents as the phase of Induction by a single occasion	For 5 weeks from weeks 16 to 21. They are suspended in this phase weekly medications	Same as in induction
