# Supplementary material for: Clinical Outcome and Prognostic Factors in Childhood Acute Lymphoblastic Leukemia—A Retrospective Cohort Study of Patients from Southern Mexico
Source: J Clin Med. 2026 Aug 31;15(17):6758. doi: 10.3390/jcm15176758 (PMC13566665; doi:10.3390/jcm15176758)
Supplement: Supplementary file 1 [file jcm-15-06758-s001.zip › Supplementary_File_S2.pdf]

Total XV protocol

Risks:

Low risk: Pre-B, DNA index >1.16, age 1-9.9 years old, TEL/AML translocation, total leukocytes <50,000. Must not have: SNC-3, testicular LLA (9;22) BCR/ABL, (1;19) E2A/PBX, MLL (11q23), EMR (+), >1% blasts at 26 days.

Standard risk: All patients with LLA cells T, and all those without low and high risk.

High risk: (9;22) BCR/ABL, induction failure or >1% blasts in AMO, EMR (+), AMO >0.1% blasts after 14 weeks postinduction, EMR re-emergence in a previously negative or EMR (+) persistent.

Before treatment: administration of intrathecal AraC, with the following schedule

1-2 years 40 mg
2-3 years 50 mg
>3 years 60 mg

Day 1: Window with HD MTX, two study groups (24 h infusion vs. 4 h infusion) and rescued with leucovorin 44 h after STARTING MTX at 50mg/m^2 for one dose, and then 15 mg/m2 every 6 h for 7 doses, and then, depending on MTX levels, administer rescues.

Subsequent intrathecal chemotherapies:

	MTX (mg)	Hydrocortisone (mg)	AraC (mg)
1-2 years	8	16	24
2-3 years	10	20	30
>3 years	12	24	36

Induction to remission (see diagram)

- Prednisone (PDN): 40mg/m^2 VO between 3 doses
- Vincristine (VCR) 1.5mg/m^2 (MAX 2mg) IV in bolo
- Daunorubicin (Dauno) 25mg/m2 IV over 1 hour
- L-Asparaginase (L-Asp) 10,000 Unidades/m^2 IM
- Cyclophosphamide 1,000mg/m^2 in 45min
- Cytarabine (AraC) 75mg/m^2 IV over 30 min
- 6-Mercaptopurine (6-MP, Purinethol): 60 mg/m^2 VO

	0	1	2	3	4	5	6	7	8	9	10	11	12	13	14	15	16	17	18	19	20	21
MTX																						
PDN*	X	X	X	X	X	X	X	X	X	X	X	X	X	X	X	X	X	X	X	X/X	X/x	x/x
VCR	X							X							X							X
Dauno	X							X														
L-ASP[a]		X		X		X		X		X		X			*		*		*			
Ciclo																						X
6-MP																						X
AMO[b]	X														X							*
QT-IT[c]	X			*											X							*

	22	23	24	25	26	27	28	29	30	31	32	33	34
AraC	X	X	X	X				X	X	X	X		
6-MP	X	X	X	X	X	X	X	X	X	X	X	X	X
AMO													X

* PDN can be switched to methylprednisolone at 20mg/m^2/day between 3 doses

[a] 3 extra doses are administered in case AMO on day 19 is found with >1% blasts, or leucocytes >50,000

[b] Day 26 AMO is performed only if extra doses of L-Asp are administered

[c] Additional QTs: only in case of CNS-2/3, traumatic PL, T-cell ALL, leucocytes >50,000, (9; 22).

CONSOLIDATION

(Must have: >1,000 leucocytes, >300 neutrophils, >50,000 platelets).

HIGH DOSES METHOTREXATE

Low risk: 2.5 g/m^2<br>Standard/High Risk: 5 g/m^2	Days 1, 15, 29, 43
QT-IT	Days 1, 15, 29, 43
6-MP: 25-50mg/m^2	Days 1-56

*Down syndrome: MTX dose 500 mg/m^2

Pre-hydration:	SG 5% + $NaHCO_3$: 200 cc/m^2/hour
	Bolus SG 5% 50cc + $NaHCO_3$ 12 mEq for low risk
	Bolus SG 5% 50cc + $NaHCO_3$ 25 mEq for High/Standard
Infusion:	10% bolus for one hour, 90% infusion for 23 hours
	IV Solutions: 100-150 cc/m^2/h + 40 mEq $NaHCO_3$
	If pH <6: bolus with 25 mEq, if pH 6-7: bolus with 12 mEq
	Acetazolamide if pH ↓: 500 mg/m^2 every 6-8 hours
	Plasma MTX measurement: From baseline: <6 h, 23 h, 42 h
Leucovorin:	15 mg/m^2 (high/std) and 10 mg/m^2 (low)
	Starts 42 h after MTX START. Every 6 hours for 5 doses
	Repeat if 42h MTX: >1.0μM, until it is <0.1μM
	If >10.0μM: Hydration, carboxypeptidase, hemoperfusion (?)
	Patients with a history of toxicity: rescues begin at 36 hours.

CONTINUATION (MAINTENANCE) INTERUM

Patients who are estimated not to tolerate high doses of MTX (sepsis, dysfunction hepatic disease, fungemia, etc.)

Once you recover, continue high-dose MTX. If this period lasts more than 4 weeks, give QT IT.

RE-INTENSIFICATION (FOR HIGH-RISK PATIENTS ONLY)

• Pre-TMO

• Patients with >0.1% EMR at week 7 of continuation, reinduction I and then this scheme

• New EMR should be taken after this scheme

• Can be repeated if EMR persists positive (>0.01%)

	Day 1	Day 2	Day 3	Day 4	Day 5	Day 6
Dexa	X	X	X	X	X	X
AraC	X	X X	X			
VP-16				X	X X	
L-ASP						X
QT-IT					X	

Dexamethasone: 20 mg/m /day between 3 doses
AraC 2000 mg/m^2 IV infusion of 3 h every 12 h, for 4 doses
Etoposide 100mg/m^2 IV in one hour, every 12 h, for 5 doses
L-Asparaginase 25,000 IM Units

MAINTENANCE (CONTINUED)
7 days after the last dose of MTX
Girls 120 weeks, boys 146 weeks

Week	High/Standard	Low
1	6MP+ASP +DEX+DOX+VCR	6MP+DEX+VCR
2	6MP+ASP	6MP+MTX
3	6MP+ASP (IT*)	6MP+MTX
4	6MP+ASP +DEX+DOX+VCR	6MP+DEX+VCR
5	6MP+ASP	6MP+MTX
6	6MP+ASP	6MP+MTX
7	(¥)REINDUCCION I +AMO+IT<br>ASP+DEX+DOX+VCR	REINDUCCION I +AMO+IT
8	REINDUCCION I<br>ASP + DOX +VCR	REINDUCCION I
9	REINDUCCION I<br>ASP+DEX+ +VCR	REINDUCCION I
10	6MP+ASP	6MP+MTX
11	6MP+ASP + DOX+VCR	6MP+MTX
12	6MP+ASP+IT	6MP+MTX+IT
13	6MP+ASP	6MP+MTX
14	6MP+ASP +DEX+DOX+VCR	6MP+DEX+VCR
15	6MP+ASP	6MP+MTX
16	6MP+ASP	6MP+MTX
17	REINDUCCION II+IT+AMO<br>ASP+DEX+ VCR	REINDUCCIOIN II+IT+AMO
18	REINDUCCION II<br>ASP+ VCR	REINDUCCION II
19	REINDUCCION II<br>ASP+DEX+**ARA-C**+VCR	REINDUCCION II
20		6MP+MTX

¥: Depending on EMR: reintensification after reinduction.
*: Very high-risk patients (bcr/abl, MLL, hypodiploidy, CNS-3, >100,000 leucocytes, LLA T-cell)
Dexamethasone (DEX): 12 mg/m^2 (high/std), 8mg/m2 (low): PO Days 1-5
Daunorubicin (DOX): 30 mg/m^2 IV in one hour, day 1
Vincristine (VCR): 2 mg/m^2 (Max. 2mg) IV bolus, day 1.
6-Mercaptopurine (6MP): 50 mg/m^2 (high/std), 75mg/m2 (low). Days 1-7
L-Asparaginase (ASP): 25,000 U/m2 IM day 1
Methotrexate (MTX): 40 mg/m^2 IM-IV day 1

RE-INDUCTION

Weeks 7-9 and 17-19
AMO in remission required

RE-INDUCTION I (high/standard)

	1	2-7	8	9-14	15	16-21
DEX	X	X	X		X	X
VCR	X		X		X	
DOX	X		X			
ASP	X		X		X	
IT, AMO	X					

RE-INDUCTION II (high/standard)

	1	2-7	8	9-14	15	16	17-20	21
DEX	X	X	X		X	X	X	X
VCR	X		X		X			
ASP	X		X		X			
ARAC					X X	X X		
IT+AMO	X							

Dexamethasone 8 mg/m^2/day in 3 doses PO
Vincristine 1.5 mg/m^2 IV bolus
Daunorubicin 30 mg/m^2 IV in 1 hour
L-Asparaginase 25,000 U/m^2 IM
Cytarabine 2,000 mg/m^2 IV infusion over 3 h, every 12 h

RE-INDUCTION II (low risk)

	1	2	3	4	5	6	7	8	9	10	11	12	13	14	15	16	17	18	19	20	21
DEX	X	X	X	X	X	X	X	X							X	X	X	X	X	X	X
VCR	X							X							X						
ASP		X		X		X		X		X		X			X		X		X		
DOX	X																				
IT	X																				

QUIMIOTERAPIA INTRATECAL

CNS-1 Patients: Weeks 7, 12, 17, 24, 32, 40, 48
Low risk with CNS-2 or >100,000 leucocytes: Weeks 7, 12, 17, 24, 28, 32, 36, 40, 44, 48
High Risk/standard: 7, 12, 17, 24, 28, 32, 36, 40, 44, 48
Very high risk: 3, 7, 12, 17, 24, 28, 32, 36, 40, 44, 48, 56, 64, 72, 80, 88, 96

MAINTENANCE (CONTINUED)
Weeks 21 onwards: (cycles are repeated every 8 weeks)

Week	High/standard	Low
21	6MP+MTX	6MP+MTX
22	6MP+MTX	6MP+MTX
23	CICLO+ARAC	6MP+MTX
24	DEX+VCR+IT	6MP+VCR+DEX+IT
25	6MP+MTX	6MP+MTX
26	6MP+MTX	6MP+MTX
27	CICLO+ARAC	6MP+MTX
28	DEX+VCR+IT	6MP+VCR+DEX (*)

*: IT only in cases with CNS2 or traumatic LP.

6-Mercaptopurine (6MP): 75 mg/m^2 PO Days 1-7
Methotrexate (MTX): 40 mg/m^2 IM o IV día 1
Cyclophosphamide (CYCLE): 300 mg/m^2 IV day 1
Cytarabine (ARAC): 300 mg/m^2 day 1
Dexamethasone (DEX): 12 mg/m^2 (high/std), 8mg/m2 (low) VO day 1-5
Vincristine (VCR): 2 mg/m^2 IV day 1

SPECIAL CONSIDERATIONS
DOWN SYNDROME

High doses of MTX: 500mg/m2
- Leucovorin starts 30 hours after the infusion, 2 doses at 30mg/m2, followed by 6 doses at 10mg/m2

MTX Maintenance:
- Same dose (40mg/m2) as far as possible. If not, it is reduced to 30→20→10. If not, give leucovorin 5mg/m2 every 6h for 4 doses

PHILADELPHIA CHROMOSOMA
Imatinib: begins on day 26 of treatment. 340 mg/m^2 (round)
During TMO it is discontinued and will restart once spinal cord function is restored.
Take with food and LOTS of WATER
Toxicity: <50,000 platelets, <500 NT, suspend and restart when NT >500. If it occurs again, restart at 50% of the dose and readjust.

CONTINGENCY PLANS

1. Induction failure. If it does not remit after induction, consolidation and reintensification: another protocol
    a. If remitting after consolidation or re-intensification: BMT
2. Hematological relapse: >25% blasts: protocol for relapses
3. Extramedullary relapse: Protocols for Relapse
    a. CNS: Re-induction of remission + IT every week 4 times. It is Continue every 3 to 4 weeks until you receive RadioTx
    b. After induction you should receive reintensification, before RadioTx
4. Refractory CNS relapse:
    a. Radiation to the skull: 24 Gy in 16 sessions in patients with <5 leucocytes before 18 months in remission
    b. Radiation to the skull: 16 Gy in 16 sessions in patients with relapse after 18 months of RCC
    c. Craniospinal radiation (24Gy to the skull in 16 sessions, 15Gy to the spine in 10 sessions) patients with >5 leucocytes within 18 months post-18 months RCC
    d. During radiation therapy, you will receive Dexa+VCR±ASP

CORRECTIONS FOR TOTAL XVI

INDUCTION TO REMISSION

- Intensive IT for high-risk CNS: 2 intrathecal tests per week for 2 weeks, and subsequent radiation assessment
- Asparaginase is changed to PEG-Asparaginase IV for one to two doses only, for long acting
- If on day 15: ≥1% → extra dose of PEG Asparaginase
- If on day 15: ≥5% → Cyclophosphamide fractionated for 3 days (gives you a 34% chance of relapse)
- 6MP will switch to 6-TG
- Imatinib change to dasatinib
- Infants are already in this protocol

CONSOLIDATION

- Low risk: 2.5 g/m² (maintain levels at 33 μM)
- High/std: According to kinetics, maintain at levels of 65 μM

MAINTENANCE

- Reinduction I/II: PEG-Asparaginase (lower dose) by L-ASP
- Reinduction II: Daunorubicin is discontinued for low risk
- The duration of treatment is 120 weeks for all
- Dexamethasone dose from week 63: 6 mg/m^2
